# Decreased preparatory activation and inattention to cues suggest lower activation of proactive cognitive control among high procrastinating students

**DOI:** 10.3758/s13415-021-00945-2

**Published:** 2021-09-08

**Authors:** Ewa Wiwatowska, Dominik Czajeczny, Jarosław M. Michałowski

**Affiliations:** 1 Department of Psychology and Law, SWPS University of Social Sciences and Humanities, Kutrzeby 10 St, 61-719 Poznań, Poland; 2 Department of Clinical Psychology, Poznań University of Medical Sciences, Poznań, Poland

**Keywords:** Attention, Cognitive control, Procrastination, Event-related potentials

## Abstract

Procrastination is a voluntary delay in completing an important task while being aware that this behavior may lead to negative outcomes. It has been shown that an increased tendency to procrastinate is associated with deficits in some aspects of cognitive control. However, none of the previous studies investigated these dysfunctions through the lenses of the Dual Mechanisms Framework, which differentiates proactive and reactive modes of control. The present study was designed to fill this gap, using behavioral and neurophysiological assessment during the completion of the AX-Continuous Performance Task (AX-CPT) by high (HP) and low (LP) procrastinating students (*N* = 139). Behavioral results indicated that HP (vs. LP) were characterized by increased attentional fluctuations (higher reaction time variability) and reduction in some indices of proactive cognitive control (lower *d*’-context and A-cue bias, but similar PBIs). Furthermore, the neurophysiological data showed that HP, compared with LP, allocated less attentional resources (lower P3b) to cues that help to predict the correct responses to upcoming probes. They also responded with reduced preparatory activity (smaller CNV) after cues presentation. The two groups did not differ in neural responses linked to conflict detection and inhibition (similar N2 and P3a). Obtained findings indicate that HP might present deficits in some cognitive functions that are essential for effective proactive control engagement, along with preserved levels of reactive cognitive control. In the present paper, we discuss the potential neural and cognitive mechanisms responsible for the observed effects.

**Supplementary Information:**

The online version contains supplementary material available at 10.3758/s13415-021-00945-2.

## Introduction

Procrastination describes the behavior of delaying tasks despite knowing that it may bring negative consequences. Increased tendency to procrastinate affects approximately 15-20% of the total population (Klingsieck, 2013) and is especially common among students (Steel, 2007). It significantly reduces their academic performance (for meta-analysis see Kim & Seo, 2015) and quality of life (Beutel et al., 2016). Although different emotional and motivational factors have been proposed as potential causes for procrastinatory behaviors, growing evidence indicates that cognitive control deficits also might contribute to the exacerbation of this problem. For example, our recent study showed that high procrastinating students present difficulties with monitoring their performance and maintaining focused attention during task completion (Michałowski et al., 2020). Moreover, Gustavson and collaborators (2015) found that procrastination is linked to lower scores in the common executive functions factor that was suggested to reflect the ability to actively maintain goal-relevant information in order to guide and control behavior (Miyake & Friedman, 2012). These procrastination-related goal management failures also have been reflected in self-report data, which showed that the tendency to delay tasks is linked to a higher frequency of cognitive slips, such as forgetting simple things or frequently making mistakes (Gustavson et al., 2014; Gustavson et al., 2015).

Overall, these findings lead to the conclusion that procrastination is associated with deficits in some aspects of cognitive control. However, this issue has not been fully explored and calls for further investigation. For example, it is unclear whether the cognitive control dysfunctions related to procrastination are more reactive or proactive, as it is defined by the Dual Mechanisms Framework (Braver, 2012). According to this concept, two distinct modes of cognitive control can be engaged during task completion: proactive control, which is associated with global, tonic activation of the cognitive system in order to anticipate upcoming events; and reactive control, which serves as a late-correction mechanism, linked to transient response to targets. Some research has shown that these two mechanisms of control might be at the ends of one dimension, with a shift towards higher proactive control resulting in lower reactive control deployment and vice versa (Boudewyn et al., 2019; Braver et al., 2009). However, some preliminary studies have indicated a possibility that proactive and reactive control might represent independent processes, which can be simultaneously applied (Gonthier et al., 2016; Mäki-Marttunen et al., 2019).

At the neural level, proactive control is associated with sustained activation in the lateral prefrontal cortex (lPFC; Jimura et al., 2010), which plays an important role in maintaining focus on task-relevant information (MacDonald et al., 2000) and anticipating incoming stimuli (Sohn et al., 2007). Reactive control is linked to transient activation of lPFC and anterior cingulate cortex (ACC; Burgess & Braver, 2010; Marini et al., 2016), which is especially active during conflict detection and inhibition of impulsive responses (Borst et al., 2014; Braver et al., 2001). Furthermore, proactive control has been suggested to be associated with the higher activity of lPFC areas to cues that help to prepare appropriate reactions to probes, while reactive control was proposed to be linked to higher lPFC activation in response to probes (Braver et al., 2009).

Proactive and reactive mechanisms of cognitive control are often studied with the use of the AX - Continuous Performance Task (AX-CPT; Cudo et al., 2018; Locke & Braver, 2008). In this task, pairs of letters appear on the screen in a cue-probe sequence. There are two types of cues (A and B) and probes (X and Y) resulting in four types of trials: AX, AY, BX, and BY (see *Methods* section for details). Trials AX are the most frequent (70%) and require a target response that is different than the response to other trials (i.e., nontarget response). Slower reactions and lower response accuracy in AY trials indicate increased proactive control engagement, as the appearance of the A-cue increases expectations and response preparation for the X-probe. Accordingly, slower and more erroneous responses in BX trials are linked to higher reactive control engagement, due to transient activation of response representation associated with the most common AX trial. Also, several other behavioral indices related to proactive control have been previously distinguished in the AX-CPT paradigm: *d*’-context, A-cue bias, and Proactive Behavioral Index (PBI). The *d*’-context and A-cue bias are measures derived from the signal detection theory (Stanislaw & Todorov, 1999); the first index reflects the ability to apply contextual information from a cue in response execution (Barch et al., 2001), and the second indicates to what extent the A cue biases individuals to execute a target response (as in AX trials) independently of the probe type (Gonthier et al., 2016). The PBI reflects the shift from the reactive to proactive mode of control from the perspective of the unidimensional approach, with higher values indicating increased proactive but decreased reactive control engagement and vice versa (Braver et al., 2009).

The AX-CPT also allows for investigating the neural correlates of reactive and proactive control processes with the use of different neuroimaging tools. One of such techniques is the event-related potential (ERP) method, which allows for the measurement of brain responses to different stimuli with high temporal precision. Several components have been identified as cognitive control indices in the AX-CPT. Increased proactive control engagement is assumed to be associated with higher amplitudes of the P3b component in response to cues (Cudo et al., 2018; Morales et al., 2015). This is a parietally distributed, positive potential, linked to allocating attentional resources to salient stimuli and updating contextual information in working memory (Kok, 2001; Lenartowicz et al., 2010; Polich, 2007). Therefore, higher amplitudes of this component might indicate greater utilization of cues in order to respond quickly and correctly to the upcoming probes (Frömer et al., 2021).

Proactive control also is reflected by more negative amplitudes of Contingent Negative Variation (CNV) preceding probe presentation (Chaillou et al., 2017; Cudo et al., 2018; Morales et al., 2015). CNV is a slowly decreasing, negative wave, which appears between cue and probe presentation and indicates both cognitive and motor response preparation as well as context maintenance (Falkenstein et al., 2003). Larger (i.e., more negative) amplitudes of this component are (similarly to P3b) linked to faster and more accurate responses (Frömer et al., 2021; Hohnsbein et al., 1998; Van Den Berg et al., 2014). Although multiple brain areas have been identified as the potential sources of CNV, numerous studies indicate the significant contribution of dorsolateral PFC (dlPFC) and ACC (Bareš et al., 2007; Gómez et al., 2003; Gómez et al., 2007; Mannarelli et al., 2015; Onoda et al., 2004; Rosahl & Knight, 1995).

Regarding reactive control engagement, it is often assumed to be reflected by more pronounced amplitudes of N2 and P3a in response to probes in AY trials (Chaillou et al., 2017; Li et al., 2018). These are frontally distributed components, which have been previously associated with ACC activity (Nieuwenhuis et al., 2003; Volpe et al., 2007). N2 is related to the detection of incongruence or conflict, for example as a result of expectations violation or competing choice alternatives (Donkers & Van Boxtel, 2004; Groom & Cragg, 2015; Nieuwenhuis et al., 2003). P3a reflects inhibition of motor response and attentional orienting towards unexpected stimuli (Enriquez-Geppert et al., 2010; Polich, 2007). Thus, larger amplitudes of these components are associated with efficient response inhibition and cognitive control in the face of conflict.

The present study aimed to investigate differences in proactive and reactive control engagement between high and low procrastinating students. We predicted that high, compared with low procrastinators, would be less effective in applying proactive control, which would be reflected by quicker and more accurate responses specifically in AY trials, decreased values of behavioral proactive control indices (*d*’-context, A-cue bias and PBI) as well as lower amplitudes of P3b and CNV after cues presentation.

We have based our hypotheses on several premises. First, procrastination has been previously linked with low goal-management skills and deficits in the common executive functions factor (Gustavson et al., 2014, 2015)—a concept that is closely related to proactive control, as it encompasses the maintenance and implementation of task-related goals (Friedman & Miyake, 2017).

Second, procrastination has been associated with decreased grey matter volume and weaker activation of dlPFC (Chen et al., 2020; Liu & Feng, 2017), as well as decreased dlPFC and ACC activity throughout longer periods in the Go/No-Go task, which measures different aspects of cognitive control (Wypych et al., 2019). The sustained character of these ACC and dlPFC functional changes, along with the structural differences within dlPFC is another argument for the possibility of lower proactive control engagement among high procrastinating individuals.

Finally, in our previous ERP study, we observed that high (vs. low) procrastinating students presented overall lower P3b amplitudes in the parametric Go/No-Go task (Michałowski et al., 2020), an effect that we suggested to reflect lower levels of sustained attention, which is essential for effective proactive control engagement. Moreover, lower P3b amplitudes in high procrastinating students were accompanied by higher reaction time variability (RTV), which might indicate fluctuations in attentional control, resulting in momentary lapses of attention and disengagement from the performing task (Esterman et al., 2013; MacDonald et al., 2009; Weissman et al., 2006). It has been suggested that increased RTV might be associated with failures in proactive cognitive control (Fassbender et al., 2014). However, this relationship has not been fully investigated yet, which is why we decided to conduct additional, correlational analyses between this measure and proactive cognitive control indices (behavioral and neurophysiological). We speculated that higher RTV would be related to lower proactive control engagement.

Regarding reactive cognitive control, we did not expect to find any differences between high and low procrastinating students, as the neural and behavioral data collected in previous studies have shown that high procrastinators have rather preserved abilities to inhibit prepotent responses and detect incongruity in the external environment (Michałowski et al., 2017; Wypych et al., 2019).

## Methods

### Questionnaires

To measure the level of academic procrastination, we used the Polish version of Aitken Procrastination Inventory (API: Aitken, 1982), which consists of 19 items with a 5-point Likert scale response format and answers ranging from 1 (False) to 5 (True). The details of the Polish adaptation procedure and its results are provided in the supplementary materials.

### Participants

Students (*N* = 1968) from different universities and colleges in Poznań completed the Polish version of API (Aitken, 1982). Of this sample, based on the standard deviation of the mean result in API, we selected 80 participants for high (scores 1 SD above the mean or higher; API ≥74; HP) and 80 subjects for low (scores 1 SD below the mean or lower; API ≤47; LP) procrastination groups. We excluded participants with psychiatric or neurological disorders as well as uncorrected vision. Of this sample, we had to exclude 21 participants: 2 participants turned out to be under psychotropic medications, 2 subjects misunderstood the instructions, 5 participants responded with too low accuracy (≥50% in AX or BY trials), 11 subjects had poor quality of EEG signal (more than 25% excluded epochs), and 1 participant prematurely ended the task. The final sample consisted of 69 participants (36 females) in the LP and 70 participants (36 females) in the HP group. The descriptive statistics of the API results for both groups are provided in the supplementary materials (see Table S1).

The study was approved by the local Ethics Committee at the SWPS University of Social Sciences and Humanities and performed in accordance with the Declaration of Helsinki. All participants signed informed consent of participation and received 80 PLN (~22 USD) at the end of the study.

### Task and procedure

**Fig. 1:**
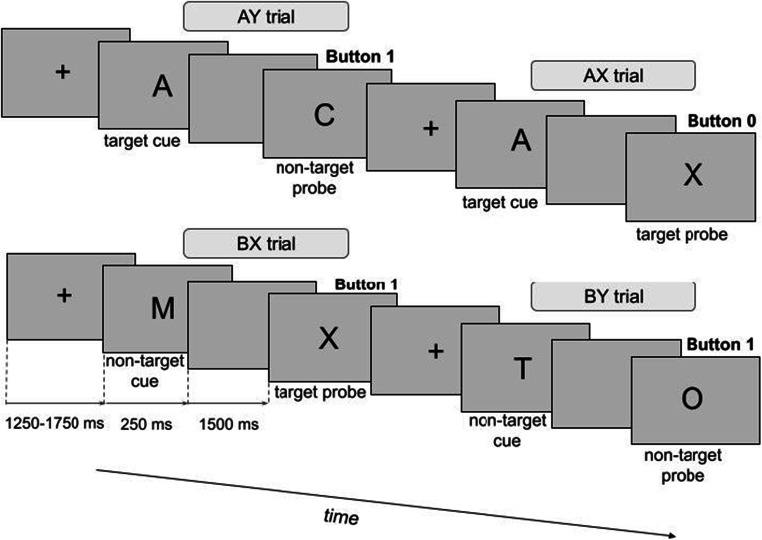
The AX - Continuous Performance Task. Pairs of letters appeared on the screen in cue-probe sequences. The letter A served as a target cue, the letter X as a target probe and letters other than A or X as nontarget probes or cues. There were four possible trial types: AX: a target cue followed by a target probe; AY: a target cue followed by a nontarget probe; BX: a nontarget cue followed by a target probe; BY: a nontarget cue followed by a nontarget probe. Participants responded to probes by pressing one of two buttons (1 or 0) on the keyboard. Trials AX occurred with 70% probability and required the response with a different button than other three trial types (each presented with 10% probability). Increased proactive control is thought to be reflected by more errors and longer reaction times in AY trials, while reactive control is linked with worse performance in BX trials.

Participants completed the AX-CPT task (Figure 1) presented on a 17” monitor placed approximately 70 cm from participants’ eyes. In the AX-CPT pairs of letters appeared on the screen in a cue-probe sequence. The letter A served as a target cue, the letter X as a target probe and letters other than A or X as nontarget cues or probes. There were four possible trial types: AX with a target cue (A) followed by a target probe (X); AY with a target cue followed by a nontarget probe (letter other than X); BX with a nontarget cue (letter other than A) followed by a target probe; and BY with a nontarget cue followed by a non-target probe. Participants had to respond to probes by pressing buttons 0 or 1 on the top row of the keyboard (with their left and right hand accordingly). Half of the participants in each group responded with 1 to probes in AX trials and with 0 to probes in other trial types, while the other half responded in the reversed manner. The response deadline was until the onset of the next cue presentation (1250-1750 ms after probes). There were 4 blocks of 100 trials with the following number of trial types in each block: AX – 70; AY – 10; BX – 10; and BY – 10. Trials were presented in a randomized order within each block. Letters were presented in black font on a grey background. The intertrial interval was randomized between 1250, 1500 and 1750 ms. Before each trial, a fixation cross appeared on the screen. A cue and a probe were presented for 250 ms. There was a 1500-ms interval between the cue offset and the onset of a probe. At the beginning of the task, there was a short training session, which could have been repeated in the case of instructions misunderstanding.

### Electrophysiological recordings and signal processing

Continuous brain activity was recorded using BrainVision Recorder and BrainAmpDC amplifier (Brain Products GmbH, Gilching, Germany) with 64 electrodes placed according to the 10-20 system. Impedances were kept below 50 kΩ and the sampling rate was 500 Hz. Data was processed offline with EEGLAB and ERPLAB toolbox (Delorme & Makeig, 2004; Lopez-Calderon & Luck, 2014) for MATLAB (The Mathworks, Inc., Natick, MA). First, the signal was filtered with 0.1-Hz high-pass and 30-Hz low-pass filters. Then, via visual inspection, we detected and interpolated noisy channels as well as manually rejected large artifacts from the signal. After that, the average reference was set and the independent component analysis was performed using the *extended runica* algorithm in EEGLAB. Visual inspection, in addition to the automatic classifier - ICLabel (Pion-Tonachini et al., 2019), was used to detect and reject components reflecting muscle and eye movements, heart activity or channel noise.

For the P3a, P3b, and N2 analyses, the data was segmented into epochs 200 ms before and 800 ms after cue or probe onset with prestimulus baseline correction. For the CNV analyses epochs were extracted from −1950- to 200-ms time window relative to probes^1^ with 200-ms precue baseline. Segments with voltages exceeding ±75 μV were rejected from averaging and participants with more than 25% artifactual epochs (11 subjects) were excluded from further analyses.

Electrodes and time windows for ERPs analyses were chosen based on previous studies (Cudo et al., 2018; Incagli et al., 2020; Morales et al., 2015) as well as the visual inspection of electrical brain activity maps (see Figure S1 in the supplementary materials) and ERP waves grand-averaged from all subjects. As a result, the following electrodes and time windows were chosen for further analyses: P3a was scored from 300 to 400 ms after the probe onset at FCz; P3b was analyzed in the time window between 400 and 600 ms after the cue onset at Pz; N2 was calculated from 200 to 300 ms after the probe presentation at FCz; CNV was scored from −200 to 0 ms before the probe onset at FCz.

### Statistical analysis

Statistical analyses were conducted with IBM SPSS Statistics 25. For each trial type, we compared rates of commission errors (incorrect button presses), omission errors (missed responses) as well as mean reaction times (RTs) for correct reactions only. RTV was calculated only for AX trials, as it was shown that this measure requires a relatively high trial number to achieve sufficient reliability (Saville et al., 2011). Other trial types were much less frequent and might have introduced some response variability resulting from other processes than failures in sustained attention. RTV was indexed as the coefficient of variation (CV), computed by dividing the standard deviation of RT by mean RT for each participant individually (Saville et al., 2011).

Regarding the behavioral indices linked to proactive control: PBI was calculated according to the formula (AY - BX)/(AY + BX) for both error rates (commission errors) and RTs in AY and BX trials; the *d*’-context was measured as the difference between z-transformed values of AX hit rate and BX commission error rate: Z(AX_hits_) - Z(BX_ER_); while the A-cue bias was calculated as the mean of z-transformed values of AX hit rate and AY commission error rate: ½*(Z[AX_hits_] + Z[AY_ER_]). The log-linear transformation was applied to all error rate and hit rate data used in the calculation of all three proactive indices in order to correct for trials with error or hit rates equal to 0 or 1 (Gonthier et al., 2016; Hautus, 1995). The transformation was applied according to the formula: error/hit rate = (number of hits/errors + 0.5) / (number of trials + 1).

To compare RTs and error rates two-way mixed ANOVAs were conducted with a group (HP vs. LP) as the between-subject factor and a trial type (AX, AY, BX, BY) as the within-group variable. Independent sample *t*-tests were conducted to measure differences between procrastination groups in RTV and proactive control indices.

For ERPs analyses, two-way mixed ANOVAs were conducted including the between-subject factor group (HP vs. LP) and the within-subject factor cue (A vs. B) for CNV and P3b analyses or a trial type (AX, AY, BX, BY) for P3a and N2 analyses. For both behavioral and electrophysiological analyses, Bonferroni and Greenhouse-Geisser corrections were applied to account for multiple comparisons and violation of sphericity assumption accordingly. Additionally, independent sample *t*-tests were run to test the differences between groups in case of a significant interaction. Two-tailed Pearson correlation analyses were performed in order to assess the relations between RTV (in AX trials) and neurophysiological and behavioral indices of proactive control.

Participants, who achieved too low accuracy (≥50%) in AX or BY trials were excluded from analyses (5 subjects).

## Results

### Behavioral data

**Fig. 2:**
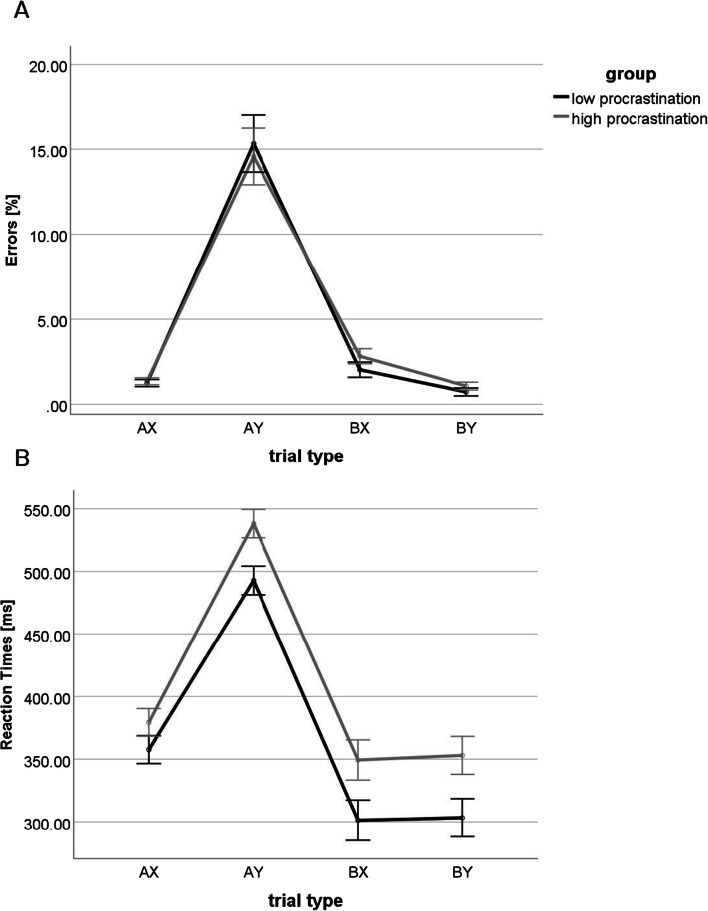
Commission error rates (**A**) and reaction times (**B**) for high and low procrastinating participants in four types of trials. Each trial consisted of a cue: type A (letter A) or B (letters other than A); and a probe: type X (letter X) or Y (letters other than X). Participants had to press one button to X probes occurring after A (i.e., AX trials) and another button in other trial types (AY or BX or BY). The AX trials were the most frequent (70% of all trials). Error bars represent one standard error

RTs and error rates are presented in Table 1 and Figure 2.

**Table 1:** Mean values (SDs) of reaction times, response accuracy and reaction-time variability (RTV) for high (HP) and low (LP) procrastinators and four trial types

Trial type	Reaction times [ms]			Commission errors [%]			Omission errors [%]		
	HP	LP	Mean	HP	LP	Mean	HP	LP	Mean
AX	379,34 (96,24)	357,57 (88,13)	368,53 (92,61)	1.35 (0.21)	1.25 (0.21)	1.30 (0.15)	2.47 (0.45)	1.67 (0.49)	2.07 (0.32)
AY	538,16 (94,93)	492,75 (94,86)	515,62 (97,26)	14.57 (1.67)	15.33 (1.68)	14.95 (1.18)	3.29 (0.58)	2.10 (0.58)	2.69 (0.41)
BX	349,35 (146,06)	301,20 (120,89)	325,45 (135,84)	2.82 (0.44)	2.03 (0.45)	2.43 (0.31)	6.36 (0.93)	6.12 (0.93)	6.24 (0.66)
BY	353,03 (129,39)	303,23 (123,81)	328,31 (128,64)	1.07 (0.23)	0.73 (0.23)	0.90 (0.16)	6.82 (1.05)	6.59 (1.06)	6.71 (0.74)

#### Reaction times

There were significant main effects of trial type for RTs (*F*(2.07; 282.85) = 502.59; *p* < 0.001; *η*_*p*_^*2*^ = 0.79). Paired comparisons revealed higher RTs for AY as compared with other trial types and increased RTs for AX in comparison to BX and BY trials (*ps* < 0.001). No differences in RT were observed between BX and BY trials (*p* > 0.1). HP responded slower than LP in all trial types (F(1,137) = 5.27; *p* = 0.023; ηp^2^ = 0.04) throughout the task.

#### Response accuracy

There were main effects of trial type for both types of error rates (F(1.11; 152.36) = 126.37; *p* < 0.001; ηp^2^ = 0.48 for commission errors; F(1.71; 234.86) = 39.87; *p* < 0.001; ηp^2^ = 0.23 for omission errors). Significant differences in commission error rates were observed between all trial pairs (*ps* < 0.05). The highest number of commission error rates was observed for AY trials, then in BX, AX, and BY trials.

The highest rate of omission rates was observed for BX and BY trials, then for AY trials and the lowest were for AX trials (*p*s < 0.05). There were no differences in omission rates between BX and BY trials (*p* > 0.1).

Regarding both omission and commission error rates, no significant group differences nor interactions were obtained (*Fs* < 1; *ps* > 0.1). Therefore, we did not confirm our hypotheses that compared with LP, HP would present decreased RTs and error rates specifically in AY trials, which would indicate lower proactive control engagement.

#### Reaction time variability

In accordance with our predictions, RTV in AX trials was higher in HP (*M* = 0.347; *SD* = 0.104) than in LP (*M* = 0.298; *SD* = 0.089) group (*t*(137) = 2.94; *p* = 0.004; *d* = 0.51). This suggests that HP show larger fluctuations in attentional control than LP.

#### Behavioral indices linked to proactive cognitive control

**Fig. 3:**
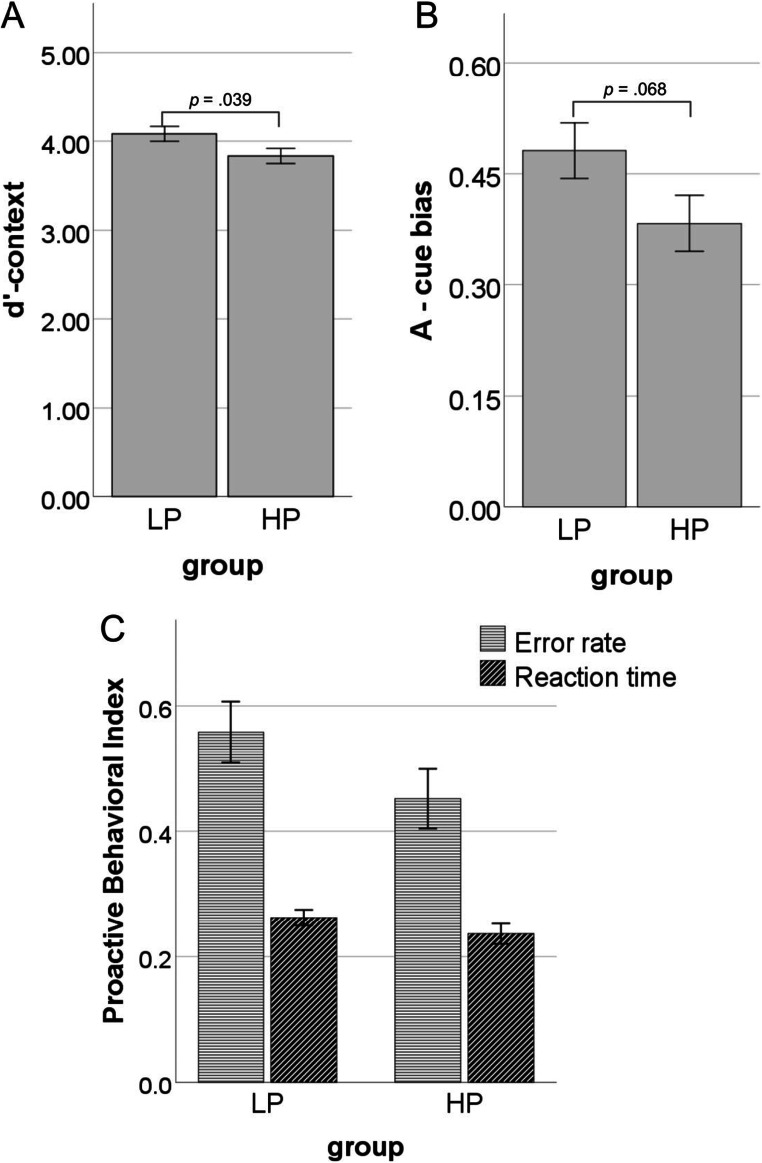
Differences between high (HP) and low (LP) procrastination groups in proactive control indices: *d*’-context (**A**), A-cue bias (**B**), Proactive Behavioral Index calculated for error rates (commission errors) and reaction times (**C**). Higher values indicate increased proactive control engagement. Error bars represent one standard error

We predicted that HP would present lower values of behavioral indices linked to proactive control engagement. In line with our predictions, *d*’-context was lower in HP than in LP group (t(137) = 2.08; *p* = 0.039; d = 0.35; Figure 3), which indicates a reduced ability to use contextual information in response execution among HP. There also was a trend-level difference in A-cue bias between groups with lower values in HP (t(137) = 1.84; *p* = 0.068; d = 0.31; Figure 3), showing that this group of participants have lower tendency to make a target response after A cues (as in AX trials) regardless of the probe type. Opposite to what we expected, there were no significant differences in PBIs between groups (*t*(137) = 1.56; *p* = 0.121; *d* = 0.26 for commission error rates; *t*(129,65) = 1.26; *p* = 0.211; *d* = 0.21 for RTs).

The results of these behavioral indices show that HP (vs. LP) present a reduced ability to use contextual information from the cues in response to probes and are less biased to make a target response (as in AX trials) after A cues, regardless of the following probe type. However, the PBI results indicate that the decreased effectiveness of proactive control in HP is not accompanied by increased reactive control.

### Electrophysiological data

#### Cue-related components

**Fig. 4:**
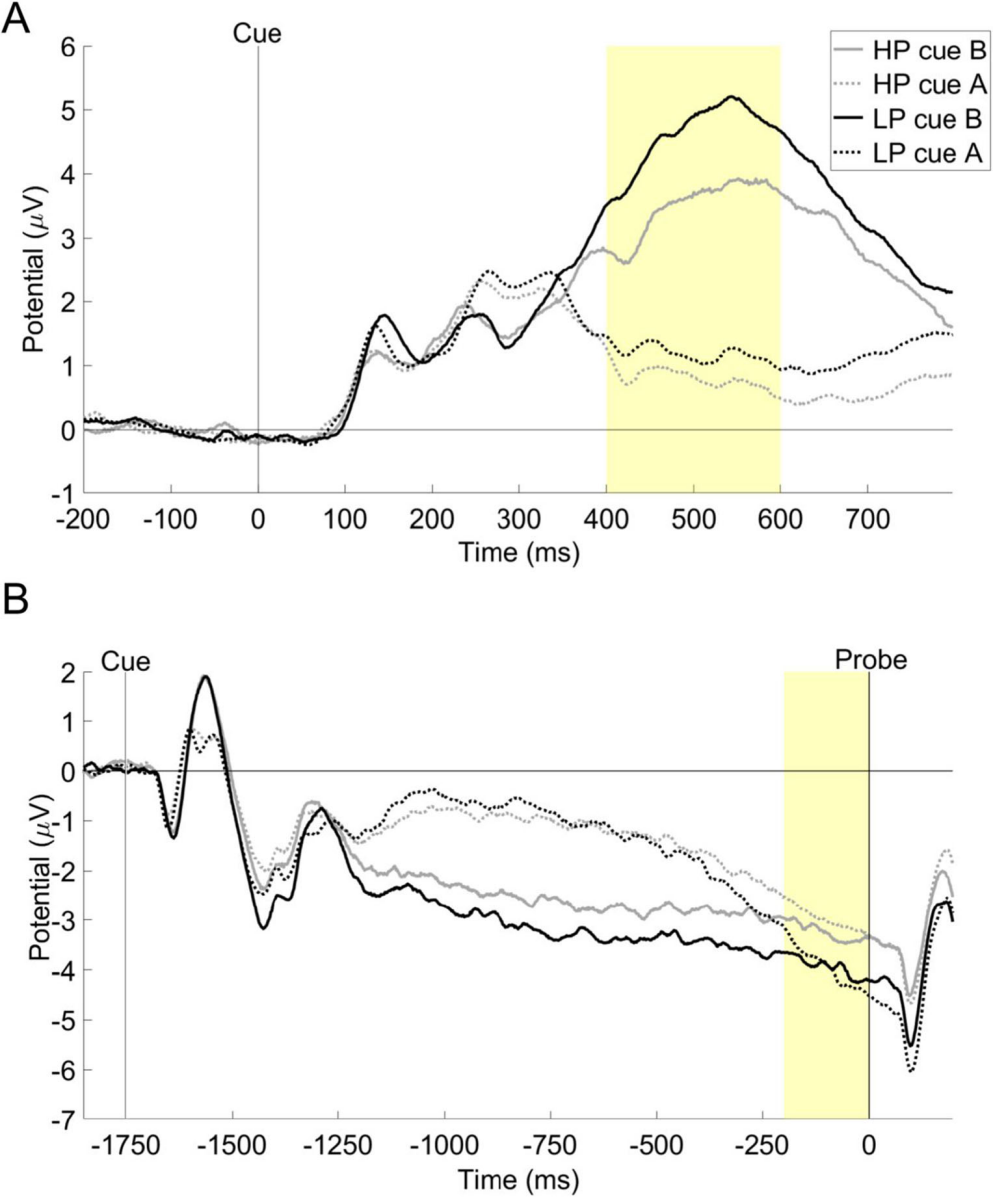
Event-related potentials (ERPs) elicited by A and B cues in the AX-Continuous Performance Task among high (HP) and low (LP) procrastination groups. (**A**) ERPs averaged over Pz with a highlighted window chosen for P3b analyses; (**B**) ERPs averaged over FCz with a highlighted window chosen for CNV analyses

The results of P3b and CNV are presented in Table 2 and Figure 4.

**Table 2:** Mean values (SDs) of P3b and CNV amplitudes elicited by A and B cues in high (HP) and low (LP) procrastinators

Cue type	P3b amplitudes [μV]		CNV amplitudes [μV]	
	HP	LP	HP	LP
A	0.88 (0.13)	1.29 (0.13)	−2.97 (0.30)	−3.94 (0.30)
B	3.53 (0.28)	4.70 (0.28)	−3.25 (0.27)	−3.94 (0.27)

P3b amplitudes were smaller in response to A vs. B cues (*F*(1,137) = 279.72; *p* < 0.001; *η*_*p*_^*2*^ = 0.67) and in HP than in LP group (*F*(1,137) = 10.10; *p* = .002; *η*_*p*_^*2*^ = 0.07) which is in accordance with our hypothesis and can be interpreted as lower attention to cues, linked with decreased proactive control employment. We also observed the significant group x cue interaction (*F*(1,137) = 4.41; *p* = .038; *η*_*p*_^*2*^ = 0.03). Post-hoc independent sample *t*-tests revealed that the differences between groups were larger in response to B cues (*t*(137) = 2.99; *p* = 0.003; *MD* = 1.17; *SE* = 0.39) compared with A cues (*t*(137) = 2.17; *p* = 0.032; *MD* = 0.41; *SE* = 0.19). As B cues are always followed by the same response irrespective of the upcoming probe, they allow for the proactive preparation of motor responses. Therefore, these results further confirm that HP present lower attention towards salient cues, which is essential for effective proactive control engagement. These findings are in line with behavioral data, which indicated lower *d*’-context among HP (see the above section).

CNV analyses confirmed our hypothesis, revealing that HP (vs. LP) presented smaller (less negative) amplitudes for both A and B cues (*F*(1,137) = 5.20; *p* = 0.024; *η*_*p*_^*2*^ = 0.04) with no significant main effect of cue or group x cue interaction (*Fs* < 1; *ps* > 0.1). This means that HP present lower preparatory activity before probes presentation. Lower CNV among HP also might contribute to reported above slower reactions to probes and lower A-cue bias, as higher preparatory activity might hinder the ability to withdraw the target response that is usually executed after A cues (see the behavioral data section).

#### Probe-related components

**Fig. 5:**
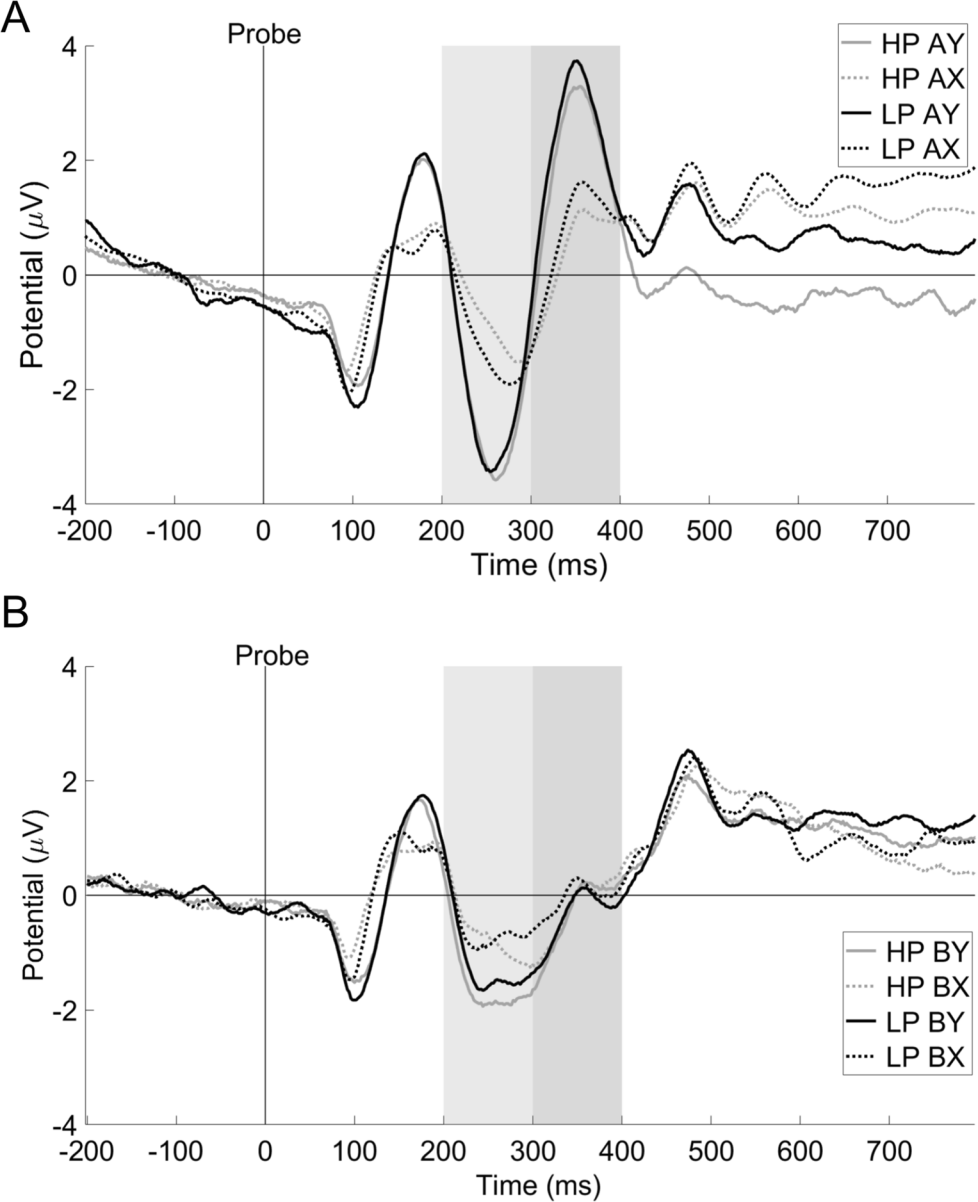
ERPs averaged over FCz, elicited by the probes in four types of trials of the AX-Continuous Performance Task high (HP) and low (LP) procrastinating participants. (**A**) ERPs in trials AX and AY. (**B**) ERPs evoked in trials BX and BY. Highlighted areas represent the time windows chosen for N2 (light grey) and P3a (dark grey) analyses

The results of N2 and P3a are presented in Table 3 and Figure 5.

**Table 3:** Mean values (SDs) of P3a and N2 amplitudes elicited by probes in high (HP) and low (LP) procrastinators in the four trial types

Trial type	P3a amplitudes [μV]		N2 amplitudes [μV]	
	HP	LP	HP	LP
AX	0.39 (2.58)	0.72 (3.48)	-0.47 (2.17)	-0.78 (2.35)
AY	1.97 (4.04)	2.29 (4.30)	-1.91 (2.68)	-1.61 (3.10)
BX	-0.25 (2.47)	-0.07 (2.87)	-0.50 (2.26)	-0.43 (2.50)
BY	-0.35 (2.61)	-0.38 (2.74)	-1.36 (2.31)	-1.05 (2.29)

N2 comparisons yielded the main effect of trial type (*F*(2.18; 298.47) = 21.70; *p* < 0.001; *η*_*p*_^*2*^ = 0.14). *Post hoc* tests showed more negative amplitudes evoked by Y probes (both in AY and BY trials) compared with those elicited by X probes (in AX and BX trials; *ps* < 0.001). There also was a trend toward bigger N2 in AY than in BY trials (*p* = 0.058). There were no significant differences in probe-related N2 amplitudes between AX and BX trials (*p* > 0.1).

P3a analyses showed the main effect of trial type (*F*(1.92; 262.58) = 48.02; *p* < 0.001; *η*_*p*_^*2*^ = 0.26). *Post hoc* tests revealed higher P3a to probes in AY trials compared with those occurring in other trial types and increased P3a to probes in AX trials when compared with those presented in BX and BY trial types (*ps* < 0.05), with no differences between BX-BY (*p* > 0.1).

We observed no main effect of group nor group x trial type interaction for both N2 and P3a (*Fs* < 2; *ps* > 0.1), which shows that there are no statistically significant differences between HP and LP in neurophysiological indices of reactive cognitive control, linked to conflict detection and inhibition.

#### Correlations with reaction time variability and proactive indices

Because RTV is supposed to reflect difficulties with sustained attention, we wanted to verify the link between this measure and proactive control indices. Confirming our predictions, increased RTV was correlated with lower *d*-context and A-cue bias as well as less pronounced P3b to B cues and CNV amplitudes. There were no significant correlations between RTV and PBIs as well as P3b in response to A cues (Table 4). This means that decreased ability to sustain attention is linked with lower utilization of cues, smaller preparatory activity before probes presentation and reduced tendency to execute a target response after A cue appearance, irrespective of the probe type. However, attentional fluctuations seem not to relate to the trade-off between performance in AY and BX trials.

**Table 4:** Correlations between reaction time variability (RTV) as well as behavioral and neurophysiological indices of proactive control

	PBI-error	PBI-RT	*d*’-context	A-cue bias	CNV-A	CNV-B	P3b-A	P3b-B
RTV	−0.092	−0.116	−0.505**	−0.254**	0.317**	0.204*	0.006	−0.269**
PBI-error		0.575**	0.308**	0.670**	−0.286**	−0.211*	0.123	0.304**
PBI-RT			0.206*	0.557**	−0.387**	−0.340**	0.013	0.365**
*d*’-context				0.443**	−0.305**	−0.236*	−0.190*	0.210*
A-cue bias					−0.374**	−0.209*	−0.058	0.271**
CNV-A						0.649**	0.058	−0.267**
CNV-B							−0.067	−0.217*
P3b-A								0.420**

**p < 0.01; *p < 0.05; RTV = reaction time variability; PBI = Proactive Behavioral Index calculated for error rate (PBI-error) or reaction times (PBI-RT); CNV = Contingent Negative Variation (higher values represent smaller - less negative - CNV); A, B - cue types.

## Discussion

We investigated the differences in proactive and reactive control between students with high and low levels of procrastination. Based on the previous research on deficits in sustained attention and goal-management failures in procrastination (Gustavson et al., 2014, 2015; Michałowski et al., 2020), we predicted that high procrastinating participants would present lower activation of proactive cognitive control than low procrastinating students. To test this hypothesis, we applied the AX-CPT paradigm along with electrophysiological measurements. Obtained results partially confirmed our predictions. Although the mean RTs, as well as error rates, in different trial types did not show a reduced proactive control pattern in high, as compared with low procrastinating participants, some of the proactive control indices were indeed lower in the high procrastination group. We also observed lower amplitudes of P3b and CNV in response to cues in high (vs. low) procrastinators, which further points out the possibility of decreased recruitment of proactive cognitive control among this group of participants. Also, we did not observe any significant differences between groups in probes-locked N2 and P3a, which indicates potentially similar reactive control engagement among high and low procrastinating subjects.

P3b reflects allocating attentional resources and updating contextual information in working memory (Polich, 2007). Smaller amplitudes of this component among high procrastinating participants might indicate lower proactive control engagement, as proper utilization of cues directs attention, reduces the number of alternative goal representations and in the end allows for more effective response preparation. Observed P3b differences between groups reached significance in response to both types of cues but were larger in response to B cues. In the AX-CPT paradigm B cues allow for the proactive preparation of the motor response, as reactions to the following probes are always the same, in contrast to A-cue trials, in which response choice is largely dependent on the following probe type (MacDonald & Carter, 2003; Mäki-Marttunen et al., 2019; Qiao et al., 2018). Therefore, larger between-group differences in P3b to B cues might indicate that high procrastinating participants allocate less attentional resources to task-relevant information, which allows for optimizing response strategy.

Along with lower P3b amplitudes, high procrastinators also showed less pronounced CNV between cue and probe presentation. Smaller amplitudes of this component might be associated with previously reported lower grey matter volume and decreased activation within dlPFC among high procrastinators (Chen et al., 2020; Liu & Feng, 2017), as this brain structure plays a significant role in behavioral control and response preparation (MacDonald et al., 2000). These structural and functional changes may significantly reduce procrastinators’ ability to maintain focus on task-relevant information and contribute to an increased tendency to reorient attention towards external or internal distractors, reducing the amount of available cognitive resources. Indeed, in previous research higher procrastination has been linked to more frequent daydreaming and intrusive thoughts (Constantin et al., 2018; Rebetez et al., 2018). Therefore, it would be interesting for future studies to further explore the associations between proactive control deficits and proneness to mind-wandering in procrastination.

Apart from lower neurophysiological indices of proactive cognitive control in high procrastinating participants, we observed no differences between groups in the probes-locked N2 and P3a amplitudes, which are the indicators of reactive cognitive control engagement. It has been shown that these components are related to inhibition abilities with larger amplitudes reflecting higher inhibitory control (Donkers & Van Boxtel, 2004; Falkenstein et al., 1999; Van Boxtel et al., 2001). N2 reflects conflict detection, while P3a is an effect of conflict resolution and motor inhibition (Enriquez-Geppert et al., 2010; Groom & Cragg, 2015). Observed results are in line with previous studies that did not demonstrate deficits in inhibitory control among high procrastinating participants (Michałowski et al., 2017; Rebetez et al., 2016; Wypych et al., 2019). However, we cannot entirely rule out the possibility of the differences between groups in reactive control engagement. It might be that the AX-CPT is better suited for investigating individual differences in proactive than reactive cognitive control. Therefore, it would be beneficial if prospective studies used different paradigms to evaluate the link between procrastination and reactive control.

Also, some researchers emphasize the role of N2 and P3a components in orienting response towards novelty and expectation violation (Nieuwenhuis et al., 2003; Schomaker & Meeter, 2014). Unfortunately, it is impossible to disentangle these processes in AX-CPT, as the appearance of a non-target probe after an A-cue both violates expectations and requires inhibition of the most frequent response associated with a target cue. Therefore, it would be interesting for future studies to further elaborate on this topic and verify whether high procrastinating participants show an attenuated response to novel stimuli that do not require motor inhibition.

Regarding behavioral measures, high procrastinators showed lower means of proactive control indices than low procrastinating subjects. However, these differences between groups reached significance only in *d*’-context along with a tendency in A-cue bias. Lower *d*’-context might reflect a decreased ability of high procrastinating subjects to use contextual information in order to adjust their behavior. This index has been previously proven to be the most reliable measure among all behavioral indices of proactive control that have been analyzed in this study (Cooper et al., 2017; Kubota et al., 2020). However, different factors can influence the *d*’-context, such as better memory for the cue or less impulsive responding. Therefore, it might indicate that high procrastinators present deficits in only some aspects of cognitive functioning that are essential for effective proactive control engagement.

Decreased A-cue bias among high procrastinators might be considered as the more direct measure of lower proactive control engagement than *d*’-context, as it measures the tendency to execute target responses for A cues independently of the probe type (Gonthier et al., 2016). Moreover, this index is a more advantageous measure than simple comparisons of error rates in AY trials, as it also takes into account the accuracy in AX trials. However, as the differences between groups in A-cue bias were at the tendency level, we should interpret this result with caution. It would certainly be beneficial to replicate this effect on a bigger sample of participants.

Although we found lower *d*’-context and A-cue bias among high (vs. low) procrastinating participants, the differences in PBIs did not reach statistical significance in the present study. The possible explanation for this pattern of results is that PBIs capture the shift from the reactive to proactive style of responding, assuming that these two modes of cognitive control are at the opposite poles of one dimension (Braver et al., 2009). Accordingly, lower PBIs would indicate smaller proactive control, but at the same time higher reactive control and vice versa. However, our findings indicate the possibility of distinct nature of these two mechanisms, as high and low procrastinating subjects differed only in some indices of proactive control engagement, with no observed differences in reactive control. These results are in line with other studies showing that these two modes of cognitive control are independent of each other and can be simultaneously applied (Gonthier et al., 2016; Mäki-Marttunen et al., 2019). In such a case, PBIs might be less sensitive to capture proactive control problems when there are no differences between groups in reactive control engagement. Nevertheless, the lack of differences between groups in PBIs is an issue worth further investigation and signals the need to interpret the obtained results with caution.

Apart from lower values of some of the proactive control indices, high procrastinators also showed slower reactions throughout the task, which might result from inattention to cues and decreased preparatory activation before probe presentation. Indeed, previous studies have shown that greater cue utilization and larger CNV amplitudes are associated with faster reactions (Brouwers et al., 2017; Hillyard, 1969; Werre et al., 2001). We also replicated our previous results regarding increased RTV among high procrastinators, which indicates difficulties in sustained attention in this group of participants (Michałowski et al., 2020). Moreover, this measure turned out to be negatively correlated with most behavioral and neurophysiological indices of proactive control, such as A-cue bias, *d*’-context, CNV and P3b to B cues. It is in line with previous findings showing that higher RTV is linked with lower proactive responding (Mäki-Marttunen et al., 2018) and reduced CNV (Doehnert et al., 2013) and indicates that this measure might be considered as another index of proactive control engagement, reflecting processes involved in sustained attention. On the contrary, we did not observe significant correlations between RTV and PBIs. The potential explanation for this effect is that the ability to sustain attention is relatively equally relevant for fast and accurate responses in both AY and BX trials. Thus, RTV might be negatively related not only to proactive but also to reactive control engagement.

Contrary to our predictions, we did not find effects of high procrastination on faster reactions or lower error rates specifically in AY trials, which would further confirm decreased proactive control engagement. However, comparing the performance on AY trials independent of other trial types might not be sensitive enough to capture more subtle differences in proactive control between groups. Although RTs should be more sensitive to between-group differences of proactive control engagement than error rate data in such easy tasks as AX-CPT, it might not be the case for comparisons of subjects that generally differ in mean RTs. For example, Locke and Braver (2008) showed that the activation of proactive control during the introduction of reward incentives was associated with more errors in AY trials but the overall faster reactions, without any specific RTs effects for AY trials. Similar findings were obtained by Mäki-Marttunen and collaborators’ (2018), who compared proactive and reactive groups of participants. Reactive subjects presented generally increased RTs, regardless of trial type, along with higher RTV. Therefore, it might be that frequent lapses of attention and slower responding are themselves indicative of reduced proactive control, despite the lack of a specific response pattern. However, these findings call for caution in drawing any definitive conclusions from this study. Future research might provide more insight into this issue, by applying different experimental paradigms to measure differences in proactive and reactive modes of control between high and low procrastinators.

According to our knowledge, this is the first study that investigated differences in proactive and reactive cognitive control activation among high and low procrastinating students. Obtained results revealed that high and low procrastinators present a similar neural response to inhibitory control and automatic conflict detection, which might indicate comparable reactive control engagement. We also observed that high as compared to low procrastinators show reduced neural activity linked to response preparation and allocation of attentional resources to task-relevant, contextual information. These neurophysiological results indicate that high procrastinators might present lower proactive control engagement than low procrastinating individuals. This is partially supported by the behavioral data, although some ambiguity in the behavioral results signals the need for caution in drawing any definitive conclusions. It would be desirable to replicate the presented findings in a correlational design study to verify whether there is a linear relationship between procrastination and proactive control recruitment. Although the comparison of extreme groups allows for capturing subtle effects in studies with a relatively small sample size, this kind of design poses some limitations. For example, it might overlook the possibility that the observed differences in cognitive control are manifested only in individuals with extreme procrastination tendencies.

Despite its limitations, the presented study provides some evidence of lower proactive control engagement in high, as compared to low procrastinating individuals. However, the associations between cognitive control recruitment and procrastination tendencies require further exploration. Future studies might take a closer look at different psychological and neuronal mechanisms that impair high procrastinators’ cognitive performance and possible solutions to overcome these problems.

## Supplementary Information

ESM 1 (DOCX 240 kb)

## Footnotes

1 In some studies, using experimental tasks with sufficiently long intertrial intervals (ISIs), early and late CNV can be distinguished (Bender et al., 2012; Funderud et al., 2012; Hart et al., 2012). However, in AX-CPT paradigms with shorter ISIs, CNV is usually scored shortly before the probe presentation (−200 or even −100 to 0 ms before probes), even though it starts to develop much earlier (Beste et al., 2011; Chaillou et al., 2017; van Wouwe et al., 2011).

## References

[CR1] Aitken ME (1982). *Personality Profile of the College Student Procrastinator*.

[CR2] Barch DM, Carter CS, Braver TS, Sabb FW, MacDonald A, Noll DC, Cohen JD (2001). Selective deficits in prefrontal cortex function in medication-naive patients with schizophrenia. Archives of General Psychiatry.

[CR3] Bareš M, Nestrašil I, Rektor I (2007). The effect of response type (motor output versus mental counting) on the intracerebral distribution of the slow cortical potentials in an externally cued (CNV) paradigm. Brain Research Bulletin.

[CR4] Bender S, Rellum T, Freitag C, Resch F, Rietschel M, Treutlein J (2012). Dopamine Inactivation Efficacy Related to Functional DAT1 and COMT Variants Influences Motor Response Evaluation. PLOS ONE.

[CR5] Beste C, Domschke K, Radenz B, Falkenstein M, Konrad C (2011). The functional 5-HT1A receptor polymorphism affects response inhibition processes in a context-dependent manner. Neuropsychologia.

[CR6] Beutel ME, Klein EM, Aufenanger S, Brähler E, Dreier M, Müller KW (2016). Procrastination, Distress and Life Satisfaction across the Age Range – A German Representative Community Study. PLOS ONE.

[CR7] Borst G, Cachia A, Vidal J, Simon G, Fischer C, Pineau A (2014). Folding of the anterior cingulate cortex partially explains inhibitory control during childhood: A longitudinal study. Developmental Cognitive Neuroscience.

[CR8] Boudewyn M, Roberts BM, Mizrak E, Ranganath C, Carter CS (2019). Prefrontal transcranial direct current stimulation (tDCS) enhances behavioral and EEG markers of proactive control. Cognitive Neuroscience.

[CR9] Braver TS (2012). The variable nature of cognitive control: A dual mechanisms framework. Trends in Cognitive Sciences.

[CR10] Braver TS, Barch DM, Gray JR, Molfese DL, Snyder A (2001). Anterior cingulate cortex and response conflict: Effects of frequency, inhibition and errors. Cerebral Cortex.

[CR11] Braver TS, Paxton JL, Locke HS, Barch DM (2009). Flexible neural mechanisms of cognitive control within human prefrontal cortex. Proceedings of the National Academy of Sciences of the United States of America.

[CR12] Brouwers S, Wiggins MW, Griffin B, Helton WS, O’Hare D (2017). The role of cue utilisation in reducing the workload in a train control task. Ergonomics.

[CR13] Burgess GC, Braver TS (2010). Neural Mechanisms of Interference Control in Working Memory: Effects of Interference Expectancy and Fluid Intelligence. PLoS ONE.

[CR14] Chaillou AC, Giersch A, Hoonakker M, Capa R, Bonnefond A (2017). Differentiating Motivational from Affective Influence of Performance-contingent Reward on Cognitive Control: The Wanting Component Enhances Both Proactive and Reactive Control. Biological Psychology.

[CR15] Chen Z, Liu P, Zhang C, Feng T (2020). Brain Morphological Dynamics of Procrastination: The Crucial Role of the Self-Control, Emotional, and Episodic Prospection Network. Cerebral Cortex.

[CR16] Constantin K, English MM, Mazmanian D (2018). Anxiety, Depression, and Procrastination Among Students: Rumination Plays a Larger Mediating Role than Worry. Journal of Rational - Emotive and Cognitive - Behavior Therapy.

[CR17] Cooper SR, Gonthier C, Barch DM, Braver TS (2017). The Role of Psychometrics in Individual Differences Research in Cognition: A Case Study of the AX-CPT. Frontiers in Psychology.

[CR18] Cudo, A., Francuz, P., Augustynowicz, P., & Stróżak, P. (2018). The Effects of Arousal and Approach Motivated Positive Affect on Cognitive Control. An ERP Study. *Frontiers in Human Neuroscience*, *12*, 320. 10.3389/fnhum.2018.00320 10.3389/fnhum.2018.00320 PMC6128242 30233339

[CR19] Delorme A, Makeig S (2004). EEGLAB: An open source toolbox for analysis of single-trial EEG dynamics including independent component analysis. Journal of Neuroscience Methods.

[CR20] Doehnert M, Brandeis D, Schneider G, Drechsler R, Steinhausen HC (2013). A neurophysiological marker of impaired preparation in an 11-year follow-up study of attention-deficit/hyperactivity disorder (ADHD). Journal of Child Psychology and Psychiatry and Allied Disciplines.

[CR21] Donkers FCL, Van Boxtel GJM (2004). The N2 in go/no-go tasks reflects conflict monitoring not response inhibition. Brain and Cognition.

[CR22] Enriquez-Geppert S, Konrad C, Pantev C, Huster RJ (2010). Conflict and inhibition differentially affect the N200/P300 complex in a combined go/nogo and stop-signal task. NeuroImage.

[CR23] Esterman M, Noonan SK, Rosenberg M, Degutis J (2013). In the zone or zoning out? Tracking behavioral and neural fluctuations during sustained attention. Cerebral Cortex.

[CR24] Falkenstein M, Hoormann J, Hohnsbein J (1999). ERP components in Go/Nogo tasks and their relation to inhibition. Acta Psychologica.

[CR25] Falkenstein M, Hoormann J, Hohnsbein J, Kleinsorge T (2003). Short-term mobilization of processing resources is revealed in the event-related potential. Psychophysiology.

[CR26] Fassbender C, Scangos K, Lesh TA, Carter CS (2014). RT distributional analysis of cognitive-control-related brain activity in first-episode schizophrenia. Cognitive, Affective and Behavioral Neuroscience.

[CR27] Friedman NP, Miyake A (2017). Unity and diversity of executive functions: Individual differences as a window on cognitive structure. Cortex.

[CR28] Frömer, R., Lin, H., Dean Wolf, C. K., Inzlicht, M., & Shenhav, A. (2021). Expectations of reward and efficacy guide cognitive control allocation. *Nature Communications*, *12*(1). 10.1038/s41467-021-21315-z 10.1038/s41467-021-21315-z PMC7884731 33589626

[CR29] Funderud I, Lindgren M, Løvstad M, Endestad T, Voytek B, Knight RT, Solbakk A-K (2012). Differential Go/NoGo Activity in Both Contingent Negative Variation and Spectral Power. PLOS ONE.

[CR30] Gómez CM, Flores A, Ledesma A (2007). Fronto-parietal networks activation during the contingent negative variation period. Brain Research Bulletin.

[CR31] Gómez CM, Marco J, Grau C (2003). Preparatory visuo-motor cortical network of the contingent negative variation estimated by current density. NeuroImage.

[CR32] Gonthier C, Braver TS, Bugg JM (2016). Dissociating proactive and reactive control in the Stroop task. Memory and Cognition.

[CR33] Groom MJ, Cragg L (2015). Differential modulation of the N2 and P3 event-related potentials by response conflict and inhibition. Brain and Cognition.

[CR34] Gustavson DE, Miyake A, Hewitt JK, Friedman NP (2014). Genetic relations among procrastination, impulsivity, and goal-management ability: Implications for the evolutionary origin of procrastination. Psychological Science.

[CR35] Gustavson DE, Miyake A, Hewitt JK, Friedman NP (2015). Understanding the cognitive and genetic underpinnings of procrastination: Evidence for shared genetic influences with goal management and executive function abilities. Journal of Experimental Psychology: General.

[CR36] Hart SJ, Lucena N, Cleary KM, Belger A, Donkers FCL (2012). Modulation of early and late event-related potentials by emotion. Frontiers in Integrative Neuroscience.

[CR37] Hautus MJ (1995). Corrections for extreme proportions and their biasing effects on estimated values of d′. Behavior Research Methods, Instruments, & Computers.

[CR38] Hillyard SA (1969). Relationships between the contingent negative variation (CNV) and reaction time. Physiology and Behavior.

[CR39] Hohnsbein J, Falkenstein M, Hoormann J (1998). Performance differences in reaction tasks are reflected in event-related brain potentials (ERPs). Ergonomics.

[CR40] Incagli F, Tarantino V, Crescentini C, Vallesi A (2020). The Effects of 8-Week Mindfulness-Based Stress Reduction Program on Cognitive Control: an EEG Study. Mindfulness.

[CR41] Jimura K, Locke HS, Braver TS (2010). Prefrontal cortex mediation of cognitive enhancement in rewarding motivational contexts. Proceedings of the National Academy of Sciences of the United States of America.

[CR42] Kim KR, Seo EH (2015). The relationship between procrastination and academic performance: A meta-analysis. Personality and Individual Differences.

[CR43] Klingsieck KB (2013). Procrastination when good things don’t come to those who wait. European Psychologist.

[CR44] Kok A (2001). On the utility of P3 amplitude as a measure of processing capacity. Psychophysiology.

[CR45] Kubota M, Hadley LV, Schaeffner S, Könen T, Meaney JA, Auyeung B (2020). Consistent use of proactive control and relation with academic achievement in childhood. Cognition.

[CR46] Lenartowicz A, Escobedo-Quiroz R, Cohen JD (2010). Updating of context in working memory: An event-related potential study. Cognitive, Affective, & Behavioral Neuroscience 2010 10:2.

[CR47] Li Y, Zhang Q, Liu F, Cui L (2018). The effect of the high-approach versus low-approach motivational positive affect on the processing stage of cognitive control: An event-related potential study. NeuroReport.

[CR48] Liu P, Feng T (2017). The overlapping brain region accounting for the relationship between procrastination and impulsivity: A voxel-based morphometry study. Neuroscience.

[CR49] Locke HS, Braver TS (2008). Motivational influences on cognitive control: Behavior, brain activation, and individual differences. Cognitive, Affective and Behavioral Neuroscience.

[CR50] Lopez-Calderon J, Luck SJ (2014). ERPLAB: an open-source toolbox for the analysis of event-related potentials. Frontiers in Human Neuroscience.

[CR51] MacDonald AW, Carter CS (2003). Event-Related fMRI Study of Context Processing in Dorsolateral Prefrontal Cortex of Patients with Schizophrenia. Journal of Abnormal Psychology.

[CR52] MacDonald AW, Cohen JD, Andrew SV, Carter CS (2000). Dissociating the role of the dorsolateral prefrontal and anterior cingulate cortex in cognitive control. Science.

[CR53] MacDonald SWS, Li SC, Bäckman L (2009). Neural Underpinnings of Within-Person Variability in Cognitive Functioning. Psychology and Aging.

[CR54] Mäki-Marttunen V, Hagen T, Aminihajibashi S, Foldal M, Stavrinou M, Halvorsen JH (2018). Ocular signatures of proactive versus reactive cognitive control in young adults. Cognitive, Affective and Behavioral Neuroscience.

[CR55] Mäki-Marttunen V, Hagen T, Espeseth T (2019). Task context load induces reactive cognitive control: An fMRI study on cortical and brain stem activity. Cognitive, Affective and Behavioral Neuroscience.

[CR56] Mannarelli, D., Pauletti, C., Grippo, A., Amantini, A., Augugliaro, V., Currà, A., … Fattapposta, F. (2015). The Role of the Right Dorsolateral Prefrontal Cortex in Phasic Alertness: Evidence from a Contingent Negative Variation and Repetitive Transcranial Magnetic Stimulation Study. *Neural Plasticity*, *2015*. 10.1155/2015/410785 10.1155/2015/410785 PMC4458283 26090234

[CR57] Marini F, Demeter E, Roberts KC, Chelazzi XL, Woldorff MG (2016). Behavioral/Cognitive Orchestrating Proactive and Reactive Mechanisms for Filtering Distracting Information: Brain-Behavior Relationships Revealed by a Mixed-Design fMRI Study. Journal of Neuroscience.

[CR58] Michałowski JM, Koziejowski W, Droździel D, Harciarek M, Wypych M (2017). Error processing deficits in academic procrastinators anticipating monetary punishment in a go/no-go study. Personality and Individual Differences.

[CR59] Michałowski, J. M., Wiwatowska, E., & Weymar, M. (2020). Brain potentials reveal reduced attention and error-processing during a monetary Go/No-Go task in procrastination. *Scientific Reports*, *10*(1). 10.1038/s41598-020-75311-2 10.1038/s41598-020-75311-2 PMC7661523 33184299

[CR60] Miyake A, Friedman NP (2012). The nature and organization of individual differences in executive functions: Four general conclusions. Current Directions in Psychological Science.

[CR61] Morales J, Yudes C, Gómez-Ariza CJ, Bajo MT (2015). Bilingualism modulates dual mechanisms of cognitive control: Evidence from ERPs. Neuropsychologia.

[CR62] Nieuwenhuis S, Yeung N, Van Den Wildenberg W, Ridderinkhof KR (2003). Electrophysiological correlates of anterior cingulate function in a go/no-go task: Effects of response conflict and trial type frequency. Cognitive, Affective and Behavioral Neuroscience.

[CR63] Onoda K, Suzuki J, Nittono H, Sakata S, Hori T (2004). LORETA analysis of CNV in time perception. International Congress Series.

[CR64] Pion-Tonachini L, Kreutz-Delgado K, Makeig S (2019). ICLabel: An automated electroencephalographic independent component classifier, dataset, and website. NeuroImage.

[CR65] Polich J (2007). Updating P300: An integrative theory of P3a and P3b. Clinical Neurophysiology.

[CR66] Qiao L, Xu L, Che X, Zhang L, Li Y, Xue G (2018). The Motivation-Based Promotion of Proactive Control: The Role of Salience Network. Frontiers in Human Neuroscience.

[CR67] Rebetez MML, Rochat L, Barsics C, Van der Linden M (2016). Procrastination as a self-regulation failure: The role of inhibition, negative affect, and gender. Personality and Individual Differences.

[CR68] Rebetez MML, Rochat L, Barsics C, Van Der Linden M (2018). Procrastination as a Self-Regulation Failure: The Role of Impulsivity and Intrusive Thoughts. Psychological Reports.

[CR69] Rosahl SK, Knight RT (1995). Role of prefrontal cortex in generation of the contingent negative variation. Cerebral Cortex.

[CR70] Saville CWN, Pawling R, Trullinger M, Daley D, Intriligator J, Klein C (2011). On the stability of instability: Optimising the reliability of intra-subject variability of reaction times. Personality and Individual Differences.

[CR71] Schomaker J, Meeter M (2014). Novelty detection is enhanced when attention is otherwise engaged: an event-related potential study. Experimental Brain Research.

[CR72] Sohn, M.-H., Albert, M. V., Jung, K., Carter, C. S., & Anderson, J. R. (2007). Anticipation of conflict monitoring in the anterior cingulate cortex and the prefrontal cortex. *Proceedings of the National Academy of Sciences of the United States of America*, *104*(25), 10330–10334. Retrieved from www.pnas.orgcgidoi10.1073pnas.0703225104 10.1073/pnas.0703225104 PMC1965513 17563353

[CR73] Stanislaw H, Todorov N (1999). Calculation of signal detection theory measures. Behavior Research Methods, Instruments, and Computers.

[CR74] Steel P (2007). The Nature of Procrastination: A Meta-Analytic and Theoretical Review of Quintessential Self-Regulatory Failure. Psychological Bulletin.

[CR75] Van Boxtel GJM, Van der Molen MW, Jennings JR, Brunia CHM (2001). A psychophysiological analysis of inhibitory motor control in the stop-signal paradigm. Biological Psychology.

[CR76] Van Den Berg B, Krebs RM, Lorist MM, Woldorff MG (2014). Utilization of reward-prospect enhances preparatory attention and reduces stimulus conflict. Cognitive, Affective and Behavioral Neuroscience.

[CR77] van Wouwe NC, Band GPH, Ridderinkhof KR (2011). Positive Affect Modulates Flexibility and Evaluative Control. Journal of Cognitive Neuroscience.

[CR78] Volpe U, Mucci A, Bucci P, Merlotti E, Galderisi S, Maj M (2007). The cortical generators of P3a and P3b: A LORETA study. Brain Research Bulletin.

[CR79] Weissman DH, Roberts KC, Visscher KM, Woldorff MG (2006). The neural bases of momentary lapses in attention. Nature Neuroscience.

[CR80] Werre PF, Mattie H, Berretty EW (2001). Contingent negative variation, extraversion, reaction time and drug effects. Personality and Individual Differences.

[CR81] Wypych M, Michałowski JM, Droździel D, Borczykowska M, Szczepanik M, Marchewka A (2019). Attenuated brain activity during error processing and punishment anticipation in procrastination – a monetary Go/No-go fMRI study. Scientific Reports.

