# Supplementary material for: Decreased preparatory activation and inattention to cues suggest lower activation of proactive cognitive control among high procrastinating students
Source: Cogn Affect Behav Neurosci. 2021 Sep 8;22(1):171–86. doi: 10.3758/s13415-021-00945-2 (PMC8791900; doi:10.3758/s13415-021-00945-2)
Supplement: Supplementary file 1 — (DOCX 240 kb) [file 13415_2021_945_MOESM1_ESM.docx]

**SUPPLEMENTARY METHODS**

**Table S1.** Aitken Procrastination Inventory - descriptive statistics for high and low procrastination groups

| Group | Mean (SD) | Skewness (SE) | Kurtosis (SE) | Minimum - Maximum |
| --- | --- | --- | --- | --- |
| Low procrastination (N = 69) | 39.67 (5.48) | -0.75 (0.29) | -0.02 (0.57) | 23.00 - 47.00 |
| High procrastination  (N = 70) | 78.97 (3.64) | 1.11 (0.29) | 1.10 (0.57) | 75.00 - 91.00 |

**
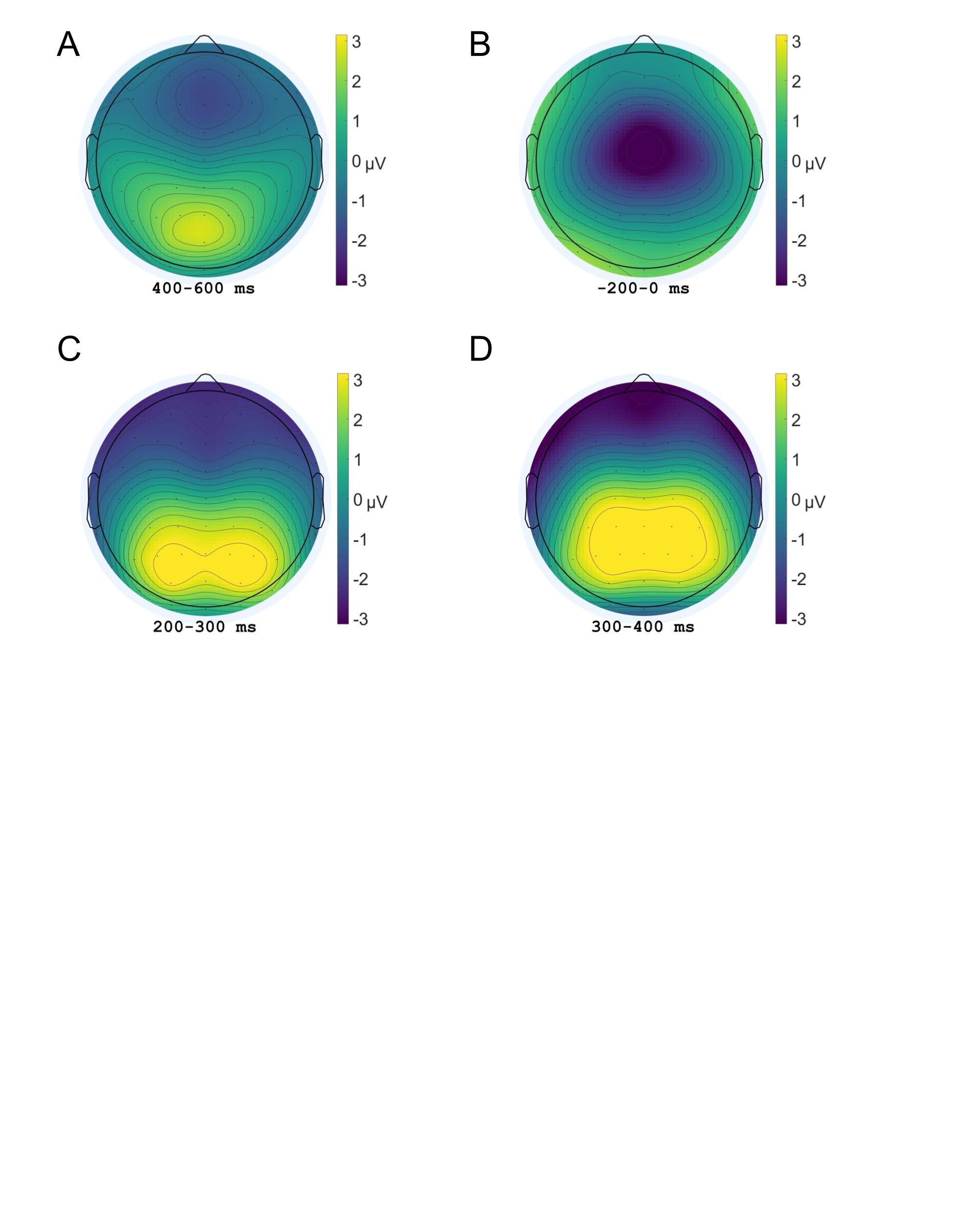
**

**Figure S1**. The maps of neurophysiological activity averaged from all subjects and trial types of the AX - Continuous Performance Task. The pictures represent the activity averaged in the following time windows chosen for the ERPs analyses: A) 400 to 600 ms after cue presentation (for P3b analysis); B) 200 to 0 ms before a probe onset (for CNV analysis); C) 200 to 300 ms after probe presentation (for N2 analysis); D) 300 to 400 ms after probe presentation (for P3a analysis)

**VALIDATION OF AITKEN PROCRASTINATION INVENTORY**

*Participants and measures*

The original version of the Aitken Procrastination Inventory (API; Aitken, 1982) was translated into Polish and then back-translated into English by two independent translators. Then, the back-translated version was compared with the original by another researcher. English translation was accepted as an equivalent of the original scale. Subsequently, the Polish questionnaire was completed by students from different universities and colleges (N = 347; 82% women) to assess the reliability and validity of the scale. The age of participants varied between 18 to 40 years old (*M*= 21.44; *SD* = 2.41).

Validity of the scale was assessed by comparing the results of API with the Polish adaptation of the Study Problem Questionnaire (SPQ; Schouwenburg, 1995; Wichrowski, 2008), which consists of 23 items organized into 3 subscales: low work discipline, fear of failure and low study interest. The scale response format is a 5-point Likert scale ranging from 1 (*highly agree*) to 5 (*highly disagree*). This questionnaire was chosen for API validation, as it encompasses three well-known correlates of procrastination: fear of failure (e.g. (Schouwenburg, 1992)), low conscientiousness (e.g. Scher & Osterman, 2002; Schouwenburg & Lay, 1995) and task aversiveness (Blunt & Pychyl, 2000; Milgram, Marshevsky, & Sadeh, 1995).

*Results*

Descriptive statistics of the API and SPQ results are presented in Table S3.

Reliability measurement showed high internal consistency of the API (Cronbach’s alpha = .89). As the original API questionnaire provides only the general result with no subscales, we assessed a single-factor model by conducting the Confirmatory Factor Analysis with AMOS 25 software. According to the modification indices, correlations were introduced between errors of items that shared variance due to similar wording (5 pairs of errors, between items: 1 and 3; 6 and 17; 6 and 18; 11 and 12; 17 and 18). The model (*X*^2^ = 339.3, *p* < .001; *df* = 147) had acceptable fit indices (CFI = .93; RMSEA = .061; SRMR = .057).

**Table S2.** Standardized factor loadings for single-factor structure of Aitken Procrastination Inventory (API)

| Item number | Standardized factor loading |
| --- | --- |
| 1 | .74 |
| 2 | .41 |
| 3 | .79 |
| 4 | .71 |
| 5 | .29 |
| 6 | .43 |
| 7 | .59 |
| 8 | .64 |
| 9 | .44 |
| 10 | .74 |
| 11 | .68 |
| 12 | .70 |
| 13 | .62 |
| 14 | .61 |
| 15 | .61 |
| 16 | .32 |
| 17 | .32 |
| 18 | .33 |
| 19 | .34 |

We observed significant correlations between API and general SPQ result (*r* = .543; *p* <.001) as well as between API and all SPQ subscales (fear of failure: *r* = .256; *p* <.001; low work discipline: *r* = .713; *p* <.001; low study interest: *r* = .264; *p* <.001).

**Table S3.** Descriptive statistics of Aitken Procrastination Inventory (API) and Study Problem Questionnaire (SPQ) with three subscales.

|  | Mean (SD) | Skewness (SE) | Kurtosis (SE) | Minimum - Maximum |
| --- | --- | --- | --- | --- |
| API | 61.37 (13.36) | -0.38 (0.13) | -0.58 (0.27) | 28 - 88 |
| SPQ | 71.20 (14.14) | -0.15 (0.13) | -0.51 (0.26) | 32 - 102 |
| SPQ: fear of failure | 31.76 (8.16) | -0.04 (0.13) | -0.69 (0.26) | 13 - 49 |
| SPQ: low work discipline | 24.27 (6.07) | -0.52 (0.13) | -0.45 (0.26) | 8 - 35 |
| SPQ: low study interest | 15.18 (4.82) | 0.32 (0.13) | -0.47 (0.26) | 6 - 29 |

**References**

Aitken, M. E. (1982). *Personality Profile of the College Student Procrastinator.* University of Pittsburgh.

Blunt, A. K., & Pychyl, T. A. (2000). Task aversiveness and procrastination: A multi-dimensional approach to task aversiveness across stages of personal projects. *Personality and Individual Differences*, *28*(1), 153–167. https://doi.org/10.1016/S0191-8869(99)00091-4

Milgram, N., Marshevsky, S., & Sadeh, C. (1995). Correlates of academic procrastination: Discomfort, task aversiveness, and task capability. *Journal of Psychology: Interdisciplinary and Applied*, *129*(2), 145–155. https://doi.org/10.1080/00223980.1995.9914954

Scher, S. J., & Osterman, N. M. (2002). Procrastination, conscientiousness, anxiety, and goals: Exploring the measurement and correlates of procrastination among school-aged children. *Psychology in the Schools*, *39*(4), 385–398. https://doi.org/10.1002/pits.10045

Schouwenburg, H. C. (1992). Procrastinators and fear of failure: an exploration of reasons for procrastination. *European Journal of Personality*, *6*(3), 225–236. https://doi.org/10.1002/per.2410060305

Schouwenburg, H. C. (1995). *Academic Procrastination*. *Procrastination and Task Avoidance*. Springer, Boston, MA. https://doi.org/10.1007/978-1-4899-0227-6_4

Schouwenburg, H. C., & Lay, C. H. (1995). Trait procrastination and the Big-five factors of personality. *Personality and Individual Differences*, *18*(4), 481–490. https://doi.org/10.1016/0191-8869(94)00176-S

Wichrowski, A. (2008). *Temperamentalne i rodzinne uwarunkowania odkładania na później spraw związanych z nauką.* University of Social Sciences and Humanities.
